# A Primary Extraskeletal Osteosarcoma of the Spleen: Rare Case Report

**DOI:** 10.3389/fonc.2022.892943

**Published:** 2022-05-02

**Authors:** Xian Pan, Han-Lu Wang, Shi-Ming Lin, Jia-Li Lin, Dan-Dan Ruan, Jian-Hui Zhang, Ting Chen, Jie-Wei Luo, Zhu-Ting Fang

**Affiliations:** ^1^ Fujian Provincial Hospital, Shengli Clinical Medical College of Fujian Medical University, Fuzhou, China; ^2^ Department of Interventional Radiology, Fujian Provincial Hospital, Fuzhou, China; ^3^ Department of Interventional Radiology, Xianyou County General Hospital, Putian, China; ^4^ Department of Traditional Chinese Medicine, Fujian Provincial Hospital, Fuzhou, China

**Keywords:** calcification, extraskeletal osteosarcoma, spleen, soft tissue tumor, osteosarcoma

## Abstract

Extraskeletal osteosarcoma is a rare malignant soft-tissue sarcoma that is difficult to diagnose. Surgery is a common treatment, although chemotherapy and radiotherapy are also used. Patients at risk of bleeding can undergo embolization combined with resection. The occurrence of primary splenic extraskeletal osteosarcoma in humans does not seem to have been reported in the literature. A 50-year-old woman who complained of pain in the left upper abdomen for 1 day was initially diagnosed with “splenic hemangioma with a high possibility of rupture and bleeding” and urgently underwent digital subtraction angiography, combined with splenic arteriography and embolization. Abdominal pain worsened 2 days postoperatively, with a hemoglobin level of 106.0 g/L. Consequently, emergency laparotomy combined with splenectomy was performed. The clinical and imaging features, pathological diagnosis, and embolization treatment of this case were analyzed retrospectively. CT of the upper abdomen revealed splenomegaly, an irregular low-density shadow in the spleen, and a flake-like calcification in the lateral margin of the left kidney. Nuclear MRI of the upper abdomen showed splenomegaly and a mass (approximately 8.4 cm × 5.7 cm × 6.3 cm) below the spleen with clear boundaries—this exhibited an uneven signal, which was slightly low in T1-weighted imaging (T1WI) and slightly high in T2-weighted imaging (T2WI). Several small cystic lesions or cystic cavities were observed in the mass, which exhibited a longer T2 signal. During the enhanced scan, the signal of the lesion showed progressive enhancement, and the enhancement range increased in the delayed phase scan, as well as a hematoma below the spleen capsule and calcification below the lesion (nodular T1WI/T2WI hypointense, approximately 3.3 cm × 3.6 cm). Postoperative biopsy pathology showed splenic soft tissue tumor: at low magnification, the multinucleated giant cells were scattered; at medium magnification, osteoclast-like multinucleated giant cells were observed; and at high magnification, lace- or grid-like tumor osteogenesis was detected. Immunohistochemistry showed that the expression of CD31, CD34, F8, s-100, desmin, SMA, and CD99 was negative, whereas the expression of β-catenin, BCL-2, SATB-2, and P16 was positive. CD68 and MDM-2 showed low expression, while 50% of the cells were positive for Ki-67 expression. No abnormal concentration of radioactivity was found on the bone scan with ^99m^Tc-MDP after the operation, further ruling out the occurrence of other bone tumors. The patient was diagnosed with primary extraskeletal osteosarcoma. It is necessary for multidisciplinary teams to diagnose malignant extraskeletal osteosarcomas.

## Introduction

Extraskeletal osteosarcoma (ESOS) is a rare malignant soft tissue sarcoma, which is considered a subtype of osteosarcoma (1). ESOS has histologic similarities to primary bone osteosarcoma except for the attachment to the bone or the periosteum. ESOS is a type of osteosarcoma that occurs in extraskeletal organs and soft tissues (2), accounting for 1% of all soft tissue sarcomas and 4% of osteogenic osteosarcomas (3). It has been reported that ESOS mostly occurs in the lower extremity, shoulder girdle, upper extremity, retroperitoneum, thigh, and hip, as well as the head and neck, gallbladder, heart, liver, kidney, bladder, breast, esophagus, prostate, spermatic cord, penis, larynx, tongue, mediastinum, lung, brain, rectum, and omentum; however, the most common location is the soft tissue of the thigh (1, 2, 4–6). ESOS is divided into primary and secondary types, and given its rarity, it is difficult to diagnose. In the present case study, we report a rare case of primary ESOS originating from the spleen, following a review of the available literature.

Clinical features lack specificity, while some patients may be asymptomatic (2, 7). While any type of primary bone tumor can potentially originate in the soft tissues, the vast majority of these extraskeletal lesions constitute osteosarcomas, chondrosarcomas, or Ewing’s sarcoma (8). ESOS originates from mesenchymal stem cells (MSCs) and can produce osteoids (9). ESOS is common in middle-aged and elderly patients and is more common in men than women (3). ESOS is a diagnosis of exclusion, because it is difficult to diagnose and needs to be combined with imaging, pathology, immunohistochemistry, and other diagnostic results to make a definite diagnosis (7). The purpose of this study was to review the characteristics of this disease and the treatment progress of ESOS and further to discuss the imaging features of ESOS in combination with this case.

## Case Presentation

On September 2, 2021, a 50-year-old woman presented with left upper abdominal pain for 1 day. Physical examination showed that the left upper abdomen was slightly distended, abdominal breathing movement was present, the left upper abdomen had tenderness without rebound pain, the liver and spleen were untouched under the ribs, Murphy’s sign was negative, and no abnormal mass was palpable. Laboratory examination showed that the white blood cell count was 21.54 × 10^9^/L (normal range: 4–10 × 10^9^/L), the percentage of neutrophils was 86.9%, hemoglobin level was 114.0 g/L (normal range: 120–160 g/L), C-reactive protein level was 60.82 mg/L (normal range: 0.5–10 mg/L), total protein level was 55.45 g/L, albumin level was 29.25 g/L, D-dimer level was 3.806 mg/L, and procalcitonin level was 0.28 ng/ml (normal range: 0–0.05 ng/ml). CT of the upper abdomen showed a small amount of fluid effusion in the bilateral pleural cavity, splenomegaly with a low-density shadow in the splenic space, and a flake-like high-density shadow of the lateral margin of the left kidney, which was considered calcification.

**Figure 1:**
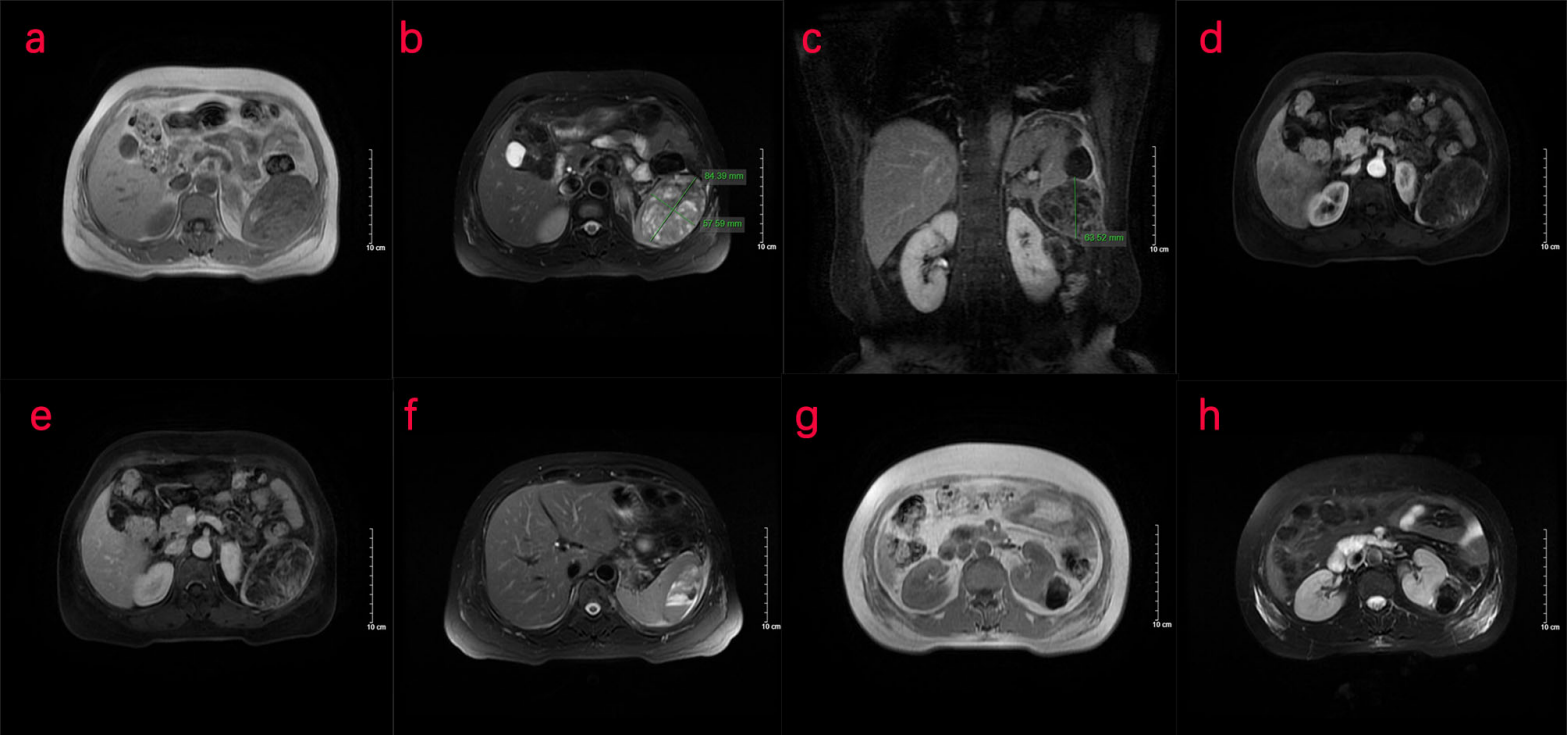
Nuclear MRI examination of the patient. **(A–H)** MRI of the upper abdomen shows an abnormal signal of the spleen. **(A–C)** The transverse position of the MRI shows that the spleen is enlarged. A mass occupying shadow, approximately 8.4 cm × 5.7 cm × 6.3 cm in size, is seen in the lower part of the spleen, with clear boundary and a slightly low signal on T1WI and slightly high signal on T2WI. Uneven signal is observed in the spleen, with small cystic cavities and a longer T2 signal. **(D, E)** MRI shows progressive enhancement patterns. **(F)** Spindle-shaped abnormal signal can be seen under the capsule of the spleen above the lesion, with a smooth edge, septa inside, mixed signals, and fluid–fluid level in the chamber, indicative of a hematoma under the capsule of the spleen. **(G, H)** A nodular T1/T2 hypointense shadow, approximately 3.3 cm × 3.6 cm in size, was seen in the lower part of the lesion compressing the left kidney nearby. T1WI, T1-weighted imaging; T2WI, T2-weighted imaging.

**Figure 2:**
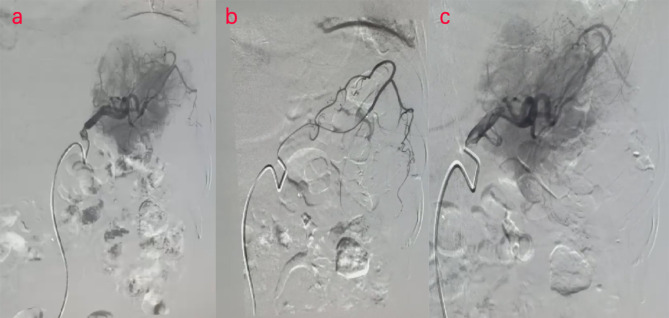
**(A–C)** Digital subtraction angiography (DSA) examination of the patient. DSA images show splenic embolism in the patient.

**Figure 3:**
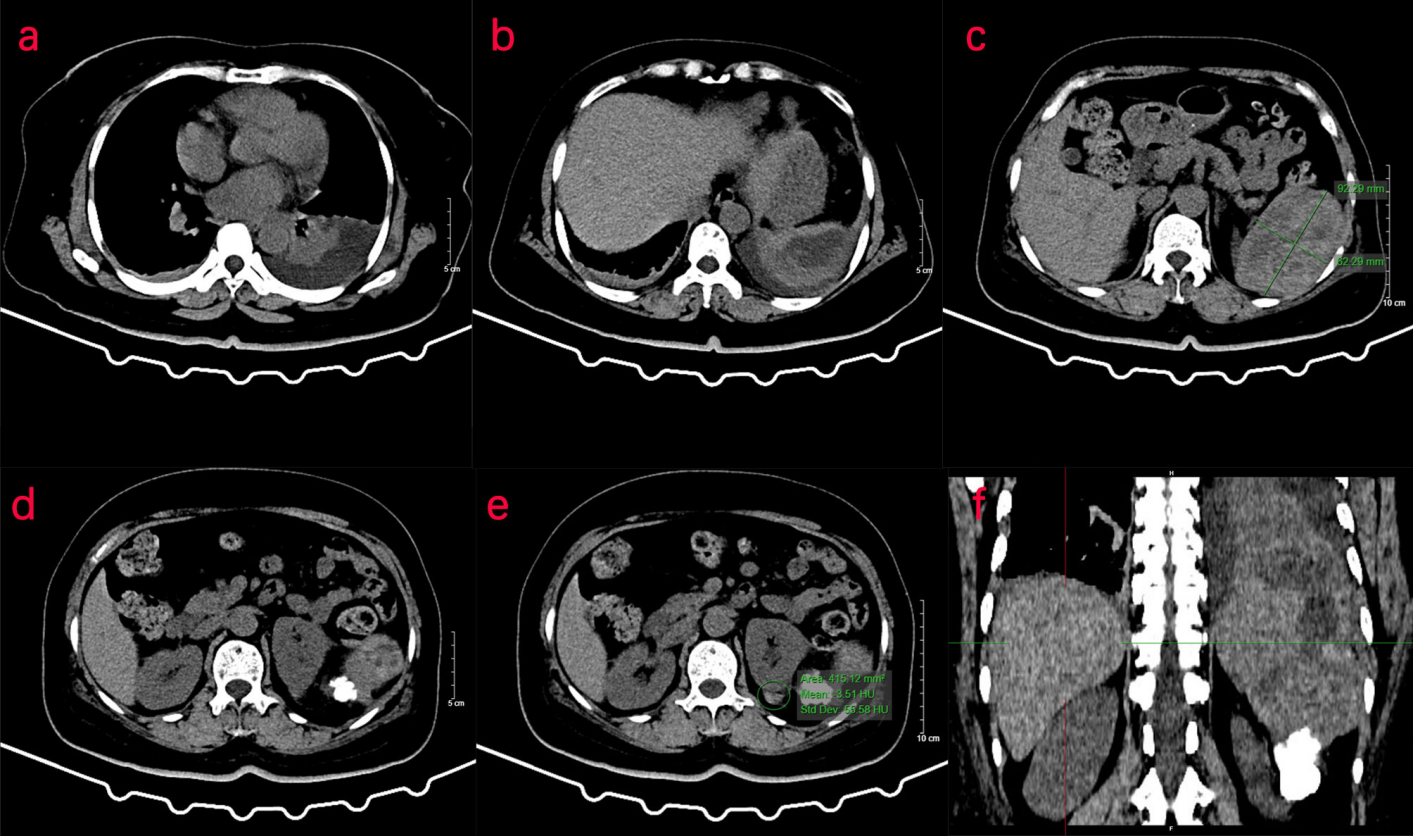
CT examination of the patient. **(A–F)** CT of the upper abdomen shows abnormal density of the spleen, and the transverse position of the CT image shows pleural effusion in the left thoracic cavity. **(C)** In the spleen, there is a mass of mixed density shadow with unclear margin, with a maximum cross-sectional area of approximately 9.2 cm × 6.1 cm. **(D, E)** The left renal margin is rough, and the density of the spindle-shaped shadow is slightly higher under the left renal capsule. **(F)** The coronal position of the CT image shows a flake-like high-density shadow in the lower margin of the spleen.

Two days later, a nuclear MRI of the upper abdomen showed splenomegaly with a mass below the spleen. The spleen occupied a space of approximately 8.4 cm × 5.7 mm × 6.3 cm, with clear boundaries. A slightly low signal was observed in T1-weighted imaging (T1WI) and a slightly high signal in T2-weighted imaging (T2WI), and the signals were uneven (**Figures 1A–E**). Small cystic degeneration or cystic spaces were observed in the mass, which showed a longer T2WI signal. The lesion showed progressive enhancement on the enhanced scan, and the enhancement range increased on the delayed scan. A spindle-shaped abnormal signal was observed above the lesion and below the splenic capsule, with smooth margins and visible separation shadows. Mixed signals and fluid–fluid levels were seen in the chamber; low, nodular T1 signal and low T2 signal were observed in association with the lesion below. The size of the mass was approximately 3.3 cm × 3.6 cm, and it compressed the adjacent left kidney. The possibility of splenic hemangioma was considered, with calcification below the lesion and splenic subcapsular hematoma formation (absorption phase) (**Figures 1F–H**). An uneven flake-like hyperintensity was observed, which was considered to be bilateral soft tissue exudation and edema of the ribs and waist. No obvious abnormalities were observed in the liver, gallbladder, or pancreas. The arc-shaped liquid signal was considered to define a small amount of fluid effusion in the left pleural cavity. After adequate examination, we combined the results with the patient’s medical history, and splenic hemangioma with ruptured bleeding was strongly considered. Digital subtraction angiography (DSA), splenic arteriography, and embolization were performed 4 days after the onset of symptoms, with the consent of the patient’s family (**Figure 2**). A super-selective catheter and gelatin sponge particles were selected for embolization, and the operation was successful. Postoperatively, anti-inflammatory, nutritional, blood transfusion, anti-infection, and other treatments were administered. After the operation, the patient experienced pain in the left upper abdomen and paroxysmal aggravation, which could not be relieved after symptomatic treatment. The hemoglobin level was 106.0 g/L. CT showed a bulk-like mixed density shadow in the spleen with poorly defined margins, a maximum cross-sectional size of approximately 9.2 cm × 6.1 cm, and hemorrhage (**Figure 3C**). A flake-like high-density shadow was seen in the lower margin of the spleen, which was considered to be a change after embolization (**Figures 3D, F**). The gallbladder shrunk and the wall thickened, which was considered chronic cholecystitis. Moreover, a spindle-shaped, slightly high-density shadow with a rough margin under the left renal capsule was observed, which was considered to be a small subcapsular hematoma of the left kidney (**Figure 3E**). Bilateral pleural thickening and arc-shaped liquid density shadows were considered to be bilateral pleural effusions (**Figures 3A, B**). Based on the above description, and considering the high possibility of active bleeding, laparotomy and splenectomy were performed under emergency general anesthesia. The surgery was successful, and the patient’s general condition was good postoperatively, without obvious discomfort.

Intraoperative abdominal exploration showed the following: approximately 300 ml of blood accumulated in the abdominal cavity; the spleen was closely adherent to the periphery; the splenic hilum and the tail of the pancreas formed a mass and were adherent; and the lower pole of the spleen was enlarged, bleeding, and adherent to the splenic flexure of the colon, with no other abnormalities observed.

Splenectomy was performed, in which the splenic flexure of the colon was fully freed from the lower pole of the spleen, and a hard mass could be palpated between the lower pole of the spleen and the left renal capsule. After dissection and ligation of the short gastric vessels, the splenic hilum was carefully separated, and the splenic artery and vein were successively dissected and doubly ligated proximally. The splenic hilum was dissected, and the spleen and the hard masses were removed.

**Figure 4:**
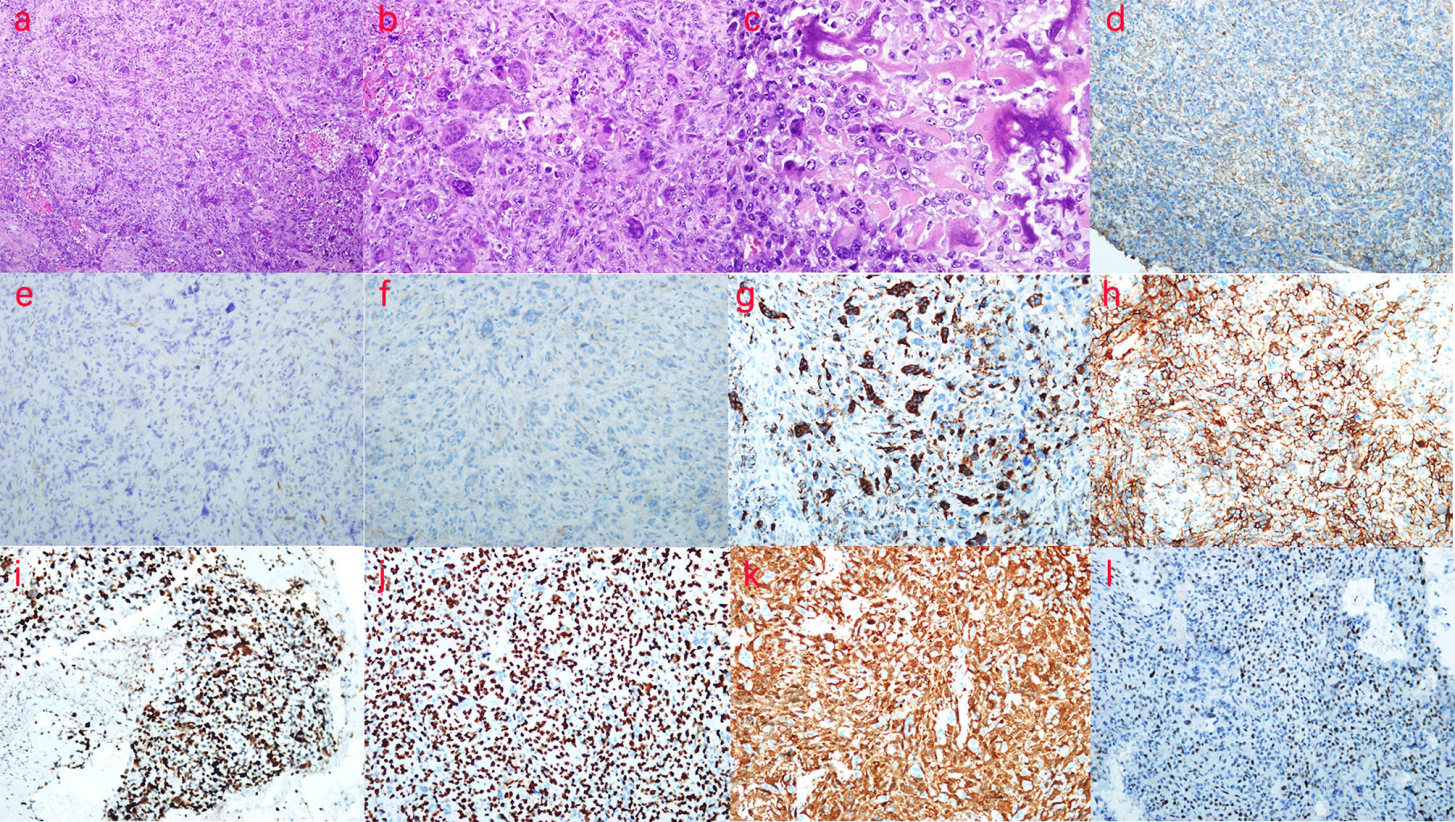
Pathological examination and immunohistochemistry (IHC) of the patient. **(A)** H&E, ×100, the tumor cells grew diffusely in sheet form, with parts arranged in a slightly weave-like manner, in which multinucleated giant cells were scattered and small areas of necrosis were seen. **(B)** H&E, ×200, osteoclast-like multinucleated giant cells were scattered in tumor cells. **(C)** H&E, ×400, lace- or grid-like tumor osteogenesis is observed. **(D)** β-Catenin (++), ×200, the tumor cell membrane in β-catenin was positive. **(E)** SMA (−),×200; **(F)** CD99 (−), ×200; **(G)** CD68 (+), ×200; positive expression of cytoplasmic CD68 was found in osteoclast-like giant cells. **(H)** BCL-2 (++), ×200; **(I)** Ki-67 (50%+), ×200; the proliferation index of Ki-67 was 50%. **(J)** SATB-2 (++), ×200; SATB-2 showed diffuse positive expression in the tumor nucleus. **(K)** P16 (++), ×200; P16 showed diffuse positive expression in the tumor; **(L)** MDM-2 (+), ×200; MDM-2 was positive in parts of the tumor cells. According to the above description, the patient was diagnosed with primary extraskeletal osteosarcoma.

The splenectomy specimen showed a spleen size of 13 cm × 8 cm × 4 cm. Bleeding was observed on the excision of the spleen. Another free mass of 5 cm × 4 cm × 3 cm with partial calcification was observed, as well as a free clot of 18 cm × 10 cm × 4 cm with partial graying that showed a splenic soft tissue tumor (**Figures 4A–C**). Under low magnification, the tumor cells grew in diffuse sheets with a slightly woven arrangement in some areas, scattered multinucleated giant cells, and small focal necrosis (**Figure 4A**). Under medium magnification, scattered osteoblast-like multinucleated giant cells were observed among tumor cells (**Figure 4B**). Under high magnification, the tumor cells were cytoplasm-rich and round/oval in shape, with many nuclear schizophrenic images and pink-stained bone-like stroma formation (tumor osteogenesis), which was lace- or grid-like, with calcium salt deposits (purple–blue) in some areas (**Figure 4C**). Immunohistochemical staining showed that the tumor cell membrane was positive for β-catenin, while the expression of SMA and CD99 was negative. Positive cytoplasmic CD68 expression was observed in osteoclast-like giant cells. BCL-2 expression was positive, and the proliferation index of Ki-67 was 50%. SATB-2 and P16 showed diffuse positive expression in the tumor nucleus. MDM-2 was positive in some tumor cells. The remaining indicators, CD31, CD34, F8, s-100, and desmin, were negatively expressed (**Figures 4D–L**). No abnormal radioactivity was found in the patient after the operation, as observed using a bone scan with ^99m^Tc-MDP, further ruling out the occurrence of other bone tumors. Accordingly, the patient was diagnosed with primary ESOS.

The patient was not treated with chemotherapy or targeted therapy for 12 months after the surgery. Multiple metastases in the lung, liver, and abdominal cavity were found at the 12-month postoperative reexamination. The patient died 13 months after the operation after being treated with lenvatinib-targeted therapy in another hospital, which was ineffective.

## Discussion

Compared to ESOS, primary osteosarcoma should be explored further. Osteogenic sarcoma (OS) is the primary solid malignancy of the bone, which produces osteoid and/or immature bone from malignant mesenchymal cells (10). The most common and important signs in radiography are the periosteal reaction and a Codman triangle (10). OS of bone attains a peak in children and early adolescents, with a second peak in adults >65 years of age (8). However, ESOS mainly occurs in middle-aged and elderly individuals (3). The etiology of osteosarcoma, which may be related to exposure to radiation, alkylating agents, or genetic variations, remains unclear. ESOS is common in the metaphyseal regions of long bones, which exhibit vigorous growth and development in adolescents, and includes the distal femur, proximal tibia, and proximal humerus (10, 11). There are two hypotheses regarding the origin of osteosarcoma: the MSC origin hypothesis and the osteoblast origin hypothesis (12). The former postulates that mutated MSCs can lead to osteosarcoma, while the latter states that osteosarcoma is caused by an osteoblast differentiation disorder (12, 13). MSCs also undergo multidirectional differentiation—fibroblasts differentiated from MSCs are usually transformed into tumor-promoting cells, which are known as cancer-associated fibroblasts (11). MSCs have the potential for multidirectional differentiation and can be transformed into other cells under specific circumstances.

Currently, there is no clear indication of ESOS etiology (2). Its pathogenesis is unclear and may be caused by multiple factors (7), including the history of trauma and radiotherapy (4, 14). According to previous reports, the mechanisms of occurrence of ESOS can be summarized under two theories. First is the metaplasia theory, which considers that the fibroblasts in the muscular interstitium are stimulated by external or internal factors, such as trauma or inflammation, and transform into osteoblasts or chondrocytes and further evolve into osteosarcoma. Most of these tumors are located in muscle tissues, and Lee et al. found that myositis ossificans caused by trauma can transform into ESOS (15). Alternatively, the tissue residue theory considers the presence of residual mesenchymal cells in the soft tissue during embryonic development, which form the bones and evolve into osteosarcoma by means of their multidirectional differentiation ability (16).

ESOS is usually treated by surgery, although chemotherapy and radiotherapy are being used. However, owing to the scarcity of cases, there is currently no standardized treatment plan. For tumors without distant metastasis, surgical resection is appropriate. For massive hemorrhage due to rupture, such as in the patient in the current report, DSA can also be used to identify the rupture and perform arterial embolization to stop the bleeding. Splenectomy should also be performed to ensure patient safety if bleeding cannot be completely stopped. According to a retrospective study of 370 ESOS cases, tumor size (HR [95% CI], 1.05 [1.02–1.07]; p < 0.001) and depth of tumor (HR [95% CI], 2.42 [1.28–4.60]; p = 0.007) were associated with systemic recurrence, and depth of tumor (HR [95% CI], 3.27 [1.20–8.21]; p = 0.02) and margin status (HR [95% CI], 5.28 [2.54–11.32]; p < 0.001) were associated with risk of local recurrence. Surgery combined with radiotherapy can reduce the risk of local recurrence (HR [95% CI], 0.46 [0.26–0.80]; p = 0.01), but chemotherapy cannot (HR [95% CI], 1.14 [0.69–2.01]; p = 0.64). Further, routine chemotherapy is not recommended for ESOS (17). For patients who cannot undergo extensive, negative margin surgical resection, radiotherapy can help provide local control of osteosarcoma. It seems to be more effective, especially in the treatment of minor or minimal residual diseases (18). This advice may also apply to ESOS. Roller et al. found that metastatic lesions mostly occurred in the lungs and the bones, and a pathological grade of the tumor affected its prognosis. Low-grade tumors had a better prognosis, and all deaths occurred in patients with high-grade tumors (19).

The primary ESOS of this patient showed a blood-rich mass of extraskeletal soft tissues with mixed signals and bleeding, but hemorrhage and hematoma are not uncommon in ESOS (3). According to the degree of hemorrhage and necrosis, both CT and MR images have varying degrees of uneven enhancement. Owing to their complex composition, the methods of enhancement also vary (8). The intratumoral calcification in this patient with ESOS was a characteristic sign, and calcification could be detected sensitively using CT images.

Reportedly, ESOS at rare sites has unusual presentations. Hunjan et al. reported a 76-year-old man with ESOS on his parietal scalp with heavy calcification. His clinical presentation resembled that of squamous cell carcinoma, and the lesion was largely asymptomatic (7). Aslan et al. reported a 69-year-old man with ESOS in the parapharyngeal region who presented with pain in the left parapharyngeal region as well as dysphagia, hoarseness, and speech disturbances with internal calcification, which was initially thought to be from the left carotid bifurcation. Therefore, it was considered a paraganglioma mass containing punctate calcified foci (1). Shankar et al. reported a 45-year-old postmenopausal woman with ESOS that occurred in the gallbladder who presented with right upper abdominal discomfort but no nausea, vomiting, weight or appetite loss, and no complications. This case was initially reported as acute calculous cholecystitis with grade 1 fatty liver on abdominal ultrasound (2).

Primary ESOS needs to be differentiated from the following diseases: i) myositis ossificans, which usually has a history of trauma and is considered to be related to abnormal repair after trauma; therefore, it is difficult to distinguish it from extraosseous osteosarcoma. Ectopic bone is formed in the soft tissue or muscle, and calcification can be observed. Lesion progression is often divided into early (<4 weeks), middle (4–8 weeks), and late (>8 weeks). Calcification is more obvious in the late stage and is more common in young men, often at the depths of soft tissues (20, 21). ii) Extraskeletal mesenchymal chondrosarcoma, which is rare, usually occurs in women and is more common in the lower extremities (22). It is characterized by dense and arc-shaped calcification, some of which can be seen as a “black pepper sign” (when calcified particles are small and dense), which is helpful in differentiating it from ESOS (22). iii) Parosteal osteosarcoma is a special type of OS, is more common in the metaphysis, and tends to grow around the diaphysis. Further, it can be connected to the cortex of the bone or exhibit a transparent gap in X-ray (23, 24). ESOS is common in the lower extremities; therefore, it must be differentiated from parosteal osteosarcoma. iv) Splenic tuberculosis is more common in patients with low immunity or HIV infection and rare in patients with normal immunity. Calcified or calcified lymph nodes are present, and common symptoms include fever, fatigue, emaciation, and splenomegaly (25). v) Splenic schistosomiasis, which may present as calcification, may occur following a history of contact with contaminated water. Further, it may present with symptoms such as anemia, leukopenia, thrombocytopenia, or hypersplenism (26). vi) Splenic hamartoma, which is rare and largely without obvious symptoms, may have cystic degeneration, calcification, and fat components. Calcification can be a mass or can be rough and is mostly found by accident (27, 28). vii) Primary angiosarcoma of the spleen, which is a malignant vascular tumor with bleeding and calcification, is characterized by splenomegaly, anemia, and weight loss (29–31).

## Conclusion

In conclusion, although calcification in soft tissues is an important sign of this disease, bleeding, degeneration, necrosis, other tumors, tuberculosis, parasitic infections, and vascular disease in soft tissues can also lead to calcification. Therefore, it is important to explore the mechanisms of osteosarcoma and ESOS. ESOS is a rare malignant soft tissue sarcoma occurring in extraskeletal organs and soft tissues, with histologic similarities to primary bone osteosarcoma except for attachment to the bone or periosteum. The etiological mechanism is unknown, and the diagnosis is difficult, which mainly depends on a multidisciplinary comprehensive diagnosis confirmed by pathological biopsy. Surgical resection remains the primary treatment option.

## Data Availability Statement

The original contributions presented in the study are included in the article/Supplementary Material. Further inquiries can be directed to the corresponding authors.

## Ethics Statement

The studies involving human participants were reviewed and approved by the Ethics Committee of Fujian Provincial Hospital. The patients/participants provided their written informed consent to participate in this study. Written informed consent was obtained from the individual(s) for the publication of any potentially identifiable images or data included in this article.

## Author Contributions

XP, H-LW, S-ML, J-LL, and D-DR acquired, analyzed, and interpreted the clinical data. XP drafted the manuscript. J-HZ, TC, J-WL, and Z-TF critically revised the manuscript. S-ML, J-WL, and Z-TF designed and supervised this study. All authors have read and approved the final manuscript.

## Funding

This work was supported by the Fujian Province Natural Science Fund Project (2020J011096, 2020J011064), the Fujian Province Medical Innovation Foundation (2021CXB001, 2019CXB004), and the Special Research Foundation of the Fujian Provincial Department of Finance (2020-822; 2021-848), China.

## Conflict of Interest

The authors declare that the research was conducted in the absence of any commercial or financial relationships that could be construed as a potential conflict of interest.

## Publisher’s Note

All claims expressed in this article are solely those of the authors and do not necessarily represent those of their affiliated organizations, or those of the publisher, the editors and the reviewers. Any product that may be evaluated in this article, or claim that may be made by its manufacturer, is not guaranteed or endorsed by the publisher.
